# Coverage of HPV Vaccination and Influencing Factors Among Female College Students in Northern China

**DOI:** 10.3390/vaccines13060598

**Published:** 2025-05-31

**Authors:** Li Yang, Chen Xing, Xue Yu, Yanrui Xu, Weibing Wang, Caiyun Chang, Qingbin Lu

**Affiliations:** 1 Jinan Center for Disease Control and Prevention, affiliated with Shandong University, Jinan 250021, China; sduyangli2012@126.com (L.Y.); 17862814126@163.com (C.X.); 19527459269@163.com (X.Y.); xyr63661@163.com (Y.X.); 2 Changqing District Disease Prevention and Control Center, Jinan 250300, China; cqjkaids@jn.shandong.cn; 3 Department of Laboratorial Science and Technology, School of Public Health, Peking University, 38 Xue-Yuan Road, Haidian District, Beijing 100871, China

**Keywords:** HPV vaccination, cervical cancer prevention, Health Belief Model, vaccine hesitancy, female college students, China

## Abstract

**Background:** Despite the significant global disease burden associated with HPV infection, the vaccination coverage among female college students in China remains suboptimal. This study aimed to examine HPV vaccination coverage, knowledge levels, and determinants influencing vaccination behavior among female college students in northern China, utilizing the Health Belief Model (HBM) as a theoretical framework. **Methods:** A cross-sectional online survey was conducted from December 2024 to January 2025, involving 4076 female students from six universities in Jinan, China. The participants were categorized into three groups: vaccinated (VG), willing-to-vaccinate (WTG), and unwilling-to-vaccinate (UTG). Data on sociodemographic characteristics, HPV knowledge, health beliefs, and vaccination behavior were analyzed using ANOVA, chi-square tests, correlation analysis, and multivariate logistic regression. **Results:** The vaccination rate was 18.11%, with 40.19% expressing willingness to vaccinate and 41.71% expressing unwillingness. Vaccinated students demonstrated higher levels of HPV knowledge (6.66 ± 2.67 compared to 4.76 ± 3.10 in the UTG, *p* < 0.001) and were predominantly from urban areas (*OR* = 0.64, *p* < 0.001). The key determinants of vaccination uptake included perceived benefits (*OR* = 1.54, *p* < 0.001), perceived barriers (*OR* = 3.34, *p* < 0.001), self-decision-making ability (OR = 1.80, *p* < 0.001), and social motivation (*OR* = 0.21, *p* < 0.001). Notably, increased knowledge was associated with vaccine hesitancy in the WTG group (*OR* = 0.45, *p* < 0.001), indicating that information overload may adversely affect decision-making processes. Structural barriers, such as cost (42.63%), safety concerns (46.59%), and misconceptions (e.g., 57.76% cited “no sexual activity” as a reason for refusal), significantly impeded vaccine uptake. **Conclusions:** The low coverage of HPV vaccination is indicative of deficiencies in knowledge, socioeconomic disparities, and cultural perceptions. Tailored interventions should focus on educational efforts to correct misconceptions, provide subsidized access to vaccines, and implement empowerment strategies that enhance self-efficacy and informed decision-making. Policymakers should incorporate these findings into national cervical cancer prevention programs to address the gap between vaccination intention and behavior among young women in China.

## 1. Introduction

Cervical cancer remains a significant global public health challenge, with an estimated 600,000 new cases and 340,000 deaths annually [1]. Human papillomavirus (HPV), a sexually transmitted DNA virus comprising over 200 identified strains, serves as the principal etiological factor in the development of cervical cancer [2]. Notably, China accounts for approximately 20% of the global burden of cervical cancer. Persistent infection with high-risk human papillomavirus (HPV) types, particularly HPV16 and HPV18, is a major etiological factor in the development of cervical cancer, accounting for approximately 70% of cervical cancer cases worldwide [3]. As the only cancer considered potentially eradicable worldwide, cervical cancer presents a unique opportunity for global health intervention. Persistent high-risk human papillomavirus (HPV) infection is the principal etiological driver of cervical carcinogenesis, thereby highlighting the essential role of HPV vaccination in cancer prevention [4]. Although HPV vaccines have demonstrated efficacy rates exceeding 90% in protecting against HPV-related diseases [5,6], vaccination coverage among female college students—a key target population due to their peak sexual activity and transitional life stage—consistently lags behind global health recommendations, especially in China [7,8]. This gap underscores the critical necessity to investigate the multifactorial determinants influencing HPV vaccination decisions among this population.

In 2016, China approved its inaugural Human Papillomavirus (HPV) vaccinations. However, the HPV vaccine has not yet been incorporated into China’s National Immunization Program (NIP) as a universally free service. Since 2022, several cities and provinces have launched pilot programs offering free HPV vaccinations, primarily targeting school-aged adolescents, particularly girls aged 13–14 years [9,10]. Despite these efforts, nationwide surveys reveal that 94.8% of female college students in China remain sexually abstinent upon entering college, with a median age of sexual debut at 19.7 years [11]. In comparison to developed countries, such as Denmark, Norway, Sweden, and the United States [12,13], where the age of sexual initiation is earlier, Chinese female college students are at the beginning stages of sexual activity, thereby increasing their risk of HPV exposure.

This delayed onset of sexual behavior and the initiation of sexual activity highlight the critical importance of targeting female college students as a key demographic for HPV vaccination initiatives. Cultural factors, particularly those rooted in traditional beliefs, may render sexual health a sensitive subject, thereby diminishing the propensity to seek vaccination. Concurrently, there exists a misconception among certain individuals that the HPV vaccine is exclusively appropriate for married women, alongside concerns regarding its potential effects on fertility.

Simultaneously, prior studies have indicated that fewer than one-third of participants were familiar with HPV and the HPV vaccine, with knowledge scores being particularly low among the Chinese population [14]. University students, in particular, demonstrated notably insufficient levels of understanding in this area [15]. This deficiency in awareness may contribute to prevalent misconceptions and vaccine hesitancy, highlighting the urgent need to address these knowledge gaps to enhance vaccination uptake. Moreover, the university setting offers an optimal environment for the implementation of health promotion initiatives, which can effectively improve HPV-related knowledge and vaccination rates. Educating young women about the significance of cancer prevention and the role of vaccination can lead to a cascading effect, ultimately benefiting future generations through increased immunization.

The Health Belief Model (HBM), which has been extensively reviewed by Glanz, Rimer, and Viswanath et al. [16], constitutes a well-established psychological framework extensively employed to elucidate health behaviors, including vaccination uptake. This model primarily comprises six dimensions: Perceived Severity, Perceived Susceptibility, Perceived Benefits, Perceived Barriers, Self-Efficacy, and Cues to Action [17]. According to the HBM, individuals’ decisions to engage in health behaviors, such as vaccination, are influenced by their perceptions of susceptibility to and severity of a health threat, alongside their beliefs regarding the benefits and barriers associated with the preventive action [18]. By addressing perceived barriers and enhancing perceived benefits and susceptibility, the HBM provides a robust theoretical foundation for designing interventions aimed at improving vaccination uptake across diverse populations. Despite its success in public health interventions, the HBM has faced criticisms, notably for being overly abstract and challenging to operationalize. While the traditional Health Belief Model (HBM) encompasses six dimensions, it does not adequately address the social, familial, and peer support systems and attitudes that impact individual health behaviors. Furthermore, it falls short of fully considering an individual’s capacity to make informed health-related decisions. Although self-efficacy and cues to action may be interconnected, researchers have modified the model to enhance its applicability by incorporating more concrete behavioral change strategies and personalized intervention measures [19]. Further expansion is essential, as it offers valuable insights into the intersection of cultural, social, and individual factors influencing vaccination decision-making.

Jinan serves as an economic and cultural hub in Shandong Province [20]. With a current permanent population of approximately 9.44 million, Jinan’s strategic location in East China serves as an important transportation hub, linking North and East China [20,21]. This strategic geographic location facilitates a rich tapestry of cultural exchanges and diversity. In the educational sector, Jinan is endowed with a wealth of higher education resources. These attributes make health trend surveys among diverse populations, especially college students in Jinan, a valuable source of insights for developing national health policies.

While numerous international studies have investigated the determinants of HPV vaccination, there is a notable deficiency of comprehensive data on HPV vaccination among female college students in China, particularly in the northern regions. Previous research on vaccination status has largely employed simplistic binary classifications, such as vaccinated versus unvaccinated [22], or willing versus unwilling to be vaccinated [23], which obscure the complex determinants influencing various behavioral subgroups. There is an inadequate differentiation between the structural or cognitive barriers encountered by those unwilling to be vaccinated and those who have actually received the vaccination. The distinction between the structural and cognitive barriers encountered by individuals hesitant to receive vaccination and those who have been vaccinated remains inadequately defined.

Furthermore, given the suboptimal penetration of HPV vaccines in China and the public’s limited awareness, this study offers a novel perspective and data on the critical factors influencing HPV vaccination status among female college students in northern China. By examining the determinants of HPV vaccination within this demographic, this study seeks to address existing geographical and cultural research gaps and to enhance the dissemination and adoption of HPV vaccines in China.

## 2. Materials and Methods

### 2.1. Study Design and Participants

A cross-sectional online design and convenience sampling method were conducted to recruit young female college students at Qilu University of Technology, Shandong Shenghan Finance and trade Vocational College, Shandong Labor Vocational and Technical College, Shandong Women’s University, Shandong University of Art and Design, and Shandong Normal University from December 2024 to January 2025, in Jinan, northern China. A stratified random sampling method was employed to ensure the representativeness of the study results, encompassing both vocational colleges and undergraduate universities.

### 2.2. Data Collection and Questionnaire

The survey was administered using Wenjuanxing (https://www.wjx.cn/, accessed on 17 December 2024), a prominent online survey platform in China recognized for its ISO-certified data security protocols. The recruitment procedure involved homeroom teachers disseminating the survey link through class WeChat groups within each university. Participation was entirely voluntary, with students being informed about the study’s objectives and the measures in place to ensure confidentiality. To prevent any form of coercion, no monetary incentives were offered. The questionnaire included the following sections: (1) Personal basic information, such as place of household registration, age, and monthly consumption level; (2) HPV vaccination behavior and willingness to vaccinate; (3) HPV-related knowledge level; (4) Motivations for HPV vaccination; (5) Attitudes towards HPV vaccination behavior; and (6) Health and sexual behavior-related content.

The participants were categorized into three groups: the vaccinated group (VG), willing-to-vaccinate group (WTG), and unwilling-to-vaccinate group (UTG). The vaccinated group refers to individuals who completed the full-dose regimen, while the latter two groups consisted of unvaccinated individuals with or without vaccination intent.

### 2.3. Ethical Approval

This study was approved by the Ethics Committee of the Jinan Center for Disease Control and Prevention (approval No.: 2024-30). Upon review by this committee, the qualifications and experience of the researchers meet the requirements for the trial; the research protocol complies with the requirements of international and domestic laws and relevant ethical norms, including the Declaration of Helsinki and the Ethical Review Measures for Biomedical Research Involving Human Subjects; the method of obtaining informed consent is appropriate; and the risks that the subjects may be subjected to are appropriate in relation to the anticipated benefits of the study.

### 2.4. HPV-Related Knowledge Level

The assessment of the participants’ knowledge levels was primarily composed of two sections, encompassing a total of 11 items. Items K1 through K8 pertained to knowledge about HPV, while items K9 through K11 addressed knowledge concerning cervical cancer. The participants were given the response options “Yes”, “No”, or “Don’t know”. A correct response was awarded one point, whereas incorrect responses or “Don’t know” selections received a score of zero. The cumulative score, termed the knowledge score, ranged from 0 to 11 points, with a maximum of 8 points allocated for HPV-related knowledge and 3 points for cervical cancer-related knowledge. Higher scores were indicative of greater knowledge levels.

### 2.5. Health Belief Model

The Health Belief Model (HBM) section addressing health beliefs related to the HPV vaccine comprised two questionnaires, which included a total of 25 items distributed across seven dimensions. Each item was evaluated using a five-point Likert scale, with response options ranging from “Strongly Disagree” to “Strongly Agree,” and corresponding scores assigned from 1 to 5. Higher scores reflected a greater level of agreement with the presented statements.

The dimensions assessed included the following: (1) **Perceived Severity**: This dimension captures the individual’s perception of the potential consequences associated with HPV infection and cervical cancer, comprising four items. (2) **Perceived Susceptibility**: This dimension pertains to the individual’s assessment of their risk of contracting HPV infection and developing cervical cancer, consisting of two items. (3) **Perceived Benefits**: This dimension evaluates the perceived advantages of receiving the HPV vaccination, including three items. (4) **Perceived Barriers**: This dimension addresses the factors that may impede individuals from obtaining the HPV vaccination, consisting of five items. (5) **Social Motivation**: This dimension encompasses the support and attitudes expressed by society, family, and peers regarding HPV vaccination among female college students, including three items. (6) **Self-Decision**: This dimension refers to the individual’s ability to make informed decisions regarding HPV vaccination, comprising four items. (7) **Self-Efficacy**: This construct pertains to an individual’s confidence in their capacity to execute behaviors associated with HPV vaccination, encompassing interpersonal communication skills and information sources, as measured by four items. See Table 1 for details.

To mitigate the influence of item quantity, the score for each dimension was computed by summing the scores of items within the dimension and dividing by the number of items.

### 2.6. Reliability and Validity Analysis

The questionnaire items in this study were developed based on pertinent literature [24,25]. The motivation and behavioral skills modules, integral components of the scale questionnaire, underwent evaluations for reliability and validity. The internal consistency of the HPV vaccination motivation scale was assessed using Cronbach’s alpha. The overall Cronbach’s alpha coefficient for the scale was 0.816, indicating satisfactory internal consistency. The Cronbach’s alpha coefficients for each dimension were 0.902, 0.940, 0.843, 0.799, and 0.861, respectively. The Kaiser–Meyer–Olkin (KMO) measure of sampling adequacy for the scale was 0.808, and Bartlett’s test of sphericity was significant (χ^2^ = 39764.809, *p* < 0.001). The cumulative variance contribution rate was 74.681%, surpassing the threshold of 60.000%, thereby indicating satisfactory structural validity of the scale. The internal consistency of the HPV vaccination behavior attitude scale was evaluated using Cronbach’s alpha, yielding an overall coefficient of 0.947. The Cronbach’s alpha coefficients for the individual dimensions were 0.952 and 0.887, respectively. The KMO value for the scale was 0.925, and Bartlett’s test of sphericity was significant (χ^2^ = 32831.660, *p* < 0.001). The cumulative variance contribution rate was 73.789%, which exceeded 60.000%.

### 2.7. Statistical Methods

Statistical analyses were performed using the Statistical Package for Social Sciences (SPSS) version 26.0 (IBM Corp., Armonk, NY, USA) and R version 4.1.2 (R Foundation for Statistical Computing, Vienna, Austria). Descriptive statistics were employed to summarize the demographic and behavioral characteristics of the participants, with continuous variables reported as means ± standard deviation (SD) and categorical variables as frequencies and percentages. Differences among the vaccinated group (VG), willing-to-vaccinate group (WTG), and unwilling-to-vaccinate group (UTG) were analyzed using one-way ANOVA for continuous data and chi-square or Fisher’s exact tests for categorical data. HPV knowledge was assessed via a questionnaire, with correct response rates and overall scores compared using one-way ANOVA. Health beliefs related to HPV vaccination were evaluated using the Health Belief Model (HBM) and one-way ANOVA, while Pearson correlation coefficients explored the relationships among HBM dimensions and vaccination status. Multivariate logistic regression identified factors (Table 2 for details) affecting HPV vaccination status, using sociodemographic characteristics, health behaviors, knowledge, and HBM scores as predictors. The model fit was verified with the likelihood ratio test, and adjusted odds ratios (*ORs*) with 95% confidence intervals (*CIs*) were reported. All the tests were two-sided with a significance level of *p* < 0.05.

### 2.8. Quality Control

The research protocol and survey questionnaire were meticulously crafted by specialists at the Jinan Center for Disease Control and Prevention. The questionnaire underwent a series of iterative discussions and revisions by a panel of experts until consensus was achieved. Prior to the formal survey implementation, a pilot test was conducted to identify and rectify potential issues. Based on feedback from the pilot test, the experts made necessary revisions and refinements to the questionnaire. Before initiating the survey, training sessions were conducted for survey personnel at the participating schools. The survey was disseminated via an online link with a specified submission deadline. The questionnaire included mandatory fields and incorporated logical validation questions; if the responses were inconsistent, the system prompted the participants to revise their answers. Furthermore, questionnaires completed in less than 90 s were excluded from the analysis. The survey was administered in Chinese, targeting participants whose native language was Chinese. The analysis included respondents with complete demographic information, thereby ensuring the validity of the survey data.

## 3. Results

### 3.1. General Participants‘ Information

A total of 4095 questionnaires were administered in this study, yielding 4076 valid responses. To ensure data integrity, logical validation checks were applied during the data collection process, resulting in a questionnaire validity rate of 99.53%. This study comprised 4076 participants, the majority of whom were of Han ethnicity (96.78%) and resided in rural areas (64.89%) (refer to Table 3 for further details). A significant proportion of the participants were under the age of 20 (88.10%), and most reported a monthly disposable income of less than RMB 2000 (84.72%). A minority (1.15%) identified with a religious affiliation, and a small percentage (3.04%) had a parent employed in the healthcare sector. This demographic profile highlights the youthful, predominantly rural, and economically moderate characteristics of the sample, with limited religious affiliation and minimal familial ties to the healthcare profession.

This investigation, encompassing 4076 participants stratified into vaccinated (VG), willing to vaccinate (WTG), and unwilling to vaccinate (UTG) cohorts, according to their HPV vaccination status, revealed significant disparities in demographic and behavioral attributes. Specifically, the VG group accounted for 18.11% (738/4076) of the participants, while the WTG and UTG groups comprised 40.19% (1638/4076) and 41.71% (1700/4076) of the sample, respectively. Notably, individuals in the VG group were more likely to reside in urban areas, have a parent employed in the healthcare sector, and exhibit higher levels of paternal education. Furthermore, this group demonstrated a greater acceptance of premarital sexual activity and had sexual activity, heightened awareness of HPV, and a more proactive approach in seeking information regarding the HPV vaccine. In contrast, the UTG group was characterized by a higher proportion of older individuals and those with elevated living expenses.

### 3.2. Level of Knowledge Regarding HPV and Related Information

**Figure 1:**
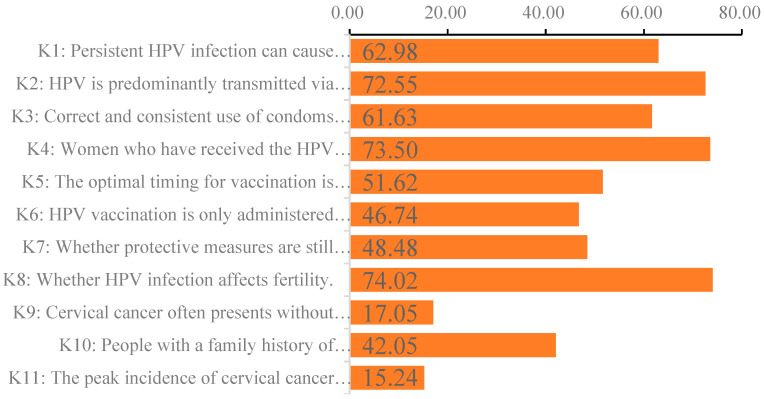
The proportion of correct responses to knowledge items for female college students.

Figure 1 illustrates the proportion of correct responses to knowledge items, with the correct response rate for each item ranging from 15.24% to 73.50%. Notably, the knowledge levels concerning cervical-cancer-related items K9, K10, and K11 were particularly low, with correct response rates of 17.05%, 42.05%, and 15.24%, respectively. The overall score for HPV-related knowledge was significantly higher in the vaccinated group (6.664 ± 2.670) compared to the group willing to be vaccinated (6.135 ± 2.704) and the group unwilling to be vaccinated (4.762 ± 3.104), with a significance level of *p* < 0.001 (refer to Table 4 for further details).

### 3.3. Analysis of Health Beliefs Associated with HPV Vaccination

Based on the Health Belief Model (HBM), significant differences in health belief dimensions were observed among female college students with different HPV vaccination statuses. Students in distinct vaccination groups exhibited statistically significant variations in perceived susceptibility (*F* = 4.865, *p* = 0.008), perceived benefits (*F* = 106.317, *p* < 0.001), perceived barriers (*F* = 155.840, *p* < 0.001), social motivation (*F* = 423.058, *p* < 0.001), self-decision-making (*F* = 391.962, *p* < 0.001), and self-efficacy (*F* = 328.42, *p* < 0.001). However, no significant difference was found in perceived severity (*F*= 0.358, *p* = 0.699) (Table 4).

**Figure 2:**
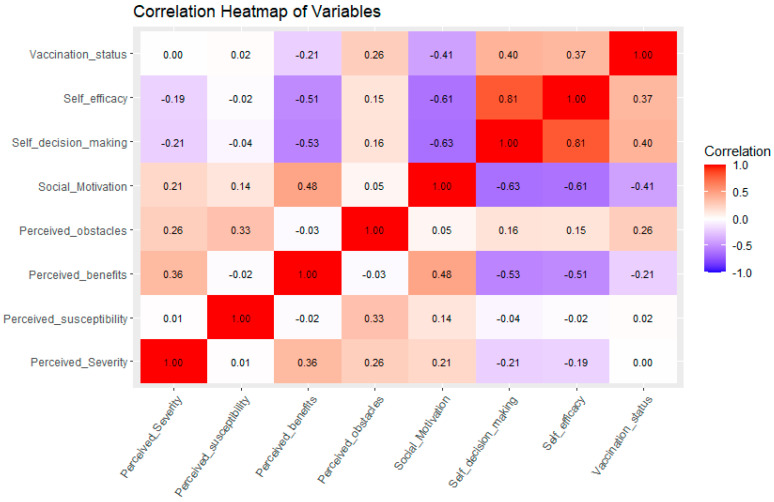
The correlation of seven dimensions and vaccination status.

The heatmap (Figure 2) depicts the correlation coefficients among the variables through a color-coded scheme, wherein red signifies positive correlations, blue denotes negative correlations, and the intensity of the color represents the correlation’s magnitude. The findings reveal that the correlation between self-efficacy and self-decision-making ability is the most pronounced (*r* = 0.81), indicating a strong reciprocal influence between these two variables. Furthermore, perceived barriers exhibit positive correlations with perceived severity and perceived susceptibility (*r* = 0.26 and *r* = 0.33, respectively), suggesting that individuals who perceive greater barriers are more inclined to believe they are susceptible to associated risks. Notably, perceived benefits are negatively correlated with perceived susceptibility (*r* = −0.02), implying that individuals with higher perceived benefits might underestimate their susceptibility in certain contexts.

Additionally, social motivation is negatively correlated with both self-efficacy and self-decision-making ability (*r* = −0.61 and *r* = −0.63, respectively), indicating that individuals with heightened social motivation may perceive diminished self-efficacy and self-decision-making ability. Vaccination status exhibited relatively low correlations with the other variables, with the highest correlation coefficient being 0.40, observed with self-decision-making. This suggests that vaccination status may be influenced by multiple factors, with self-decision-making potentially playing a more significant role.

### 3.4. Multivariate Analysis of Factors Influencing HPV Vaccination Status

To explore the factors influencing HPV vaccination status, a multivariate logistic regression analysis was conducted, with HPV vaccination status as the dependent variable (unwilling-to-vaccinate group [UTG] = 0, willing-to-vaccinate group [WTG] = 1, vaccinated group [VG] = 2). The independent variables included sociodemographic characteristics (ethnicity, domicile, religion, monthly disposable income, whether one of the parents is a healthcare worker, paternal educational level, and whether there is a family or friend history of cervical cancer), health and sexual behaviors (acceptance of premarital sex, whether the participant has ever engaged in sexual activity, whether they have received sex education, whether they have heard of HPV, whether they have actively searched for or consulted about the HPV vaccine, and knowledge level of HPV), and scores from the Health Belief Model (HBM) dimensions. The variable assignments are detailed in Table 4.

The likelihood ratio test for the model indicated a good fit (χ² = 1731.812, *p* < 0.001), confirming the model’s validity. When comparing the vaccinated group (VG) with the unwilling-to-vaccinate group (UTG), the regression results reveal that students residing in urban areas (*OR* = 0.64, *95% CI:* 0.49–0.82, *p* < 0.001), those aged ≤20 years (*OR* = 0.38, *95% CI:* 0.28–0.51, *p* < 0.001), those with lower paternal education levels (e.g., junior high school or below: *OR* = 0.36, *95% CI:* 0.16–0.82, *p* = 0.01; high school/vocational school: *OR* = 0.41, *95% CI:* 0.18–0.91, *p* = 0.03), those with a family history of cervical cancer (*OR* = 0.37, *95% CI:* 0.18–0.76, *p* = 0.01), those who had engaged in sexual activity *(OR* = 0.57, *95% CI:* 0.39–0.85, *p* = 0.01), those who had actively searched for or consulted about the HPV vaccine (*OR* = 0.45, *95% CI*: 0.34–0.59, *p* < 0.001), those with higher perceived benefits of vaccination (*OR* = 1.54, *95% CI*: 1.27–1.86, *p* < 0.001), those with weaker social motivation (*OR* = 0.21, *95% CI*: 0.17–0.26, *p* < 0.001), and those with stronger self-decision-making abilities (*OR* = 1.8, *95% CI*: 1.38–2.35, *p* < 0.001) were significantly more likely to be vaccinated (Table S1).

When comparing the willing-to-vaccinate group (WTG) with the unwilling-to-vaccinate group (UTG), the regression results show that students aged ≤20 years (*OR* = 1.43, *95% CI*: 1.11–1.85, *p* = 0.01), those with a monthly disposable income of RMB 1500–2000 (*OR* = 1.25, *95% CI*: 0.85–1.86, *p* = 0.23), those with parents working in healthcare (*OR* = 1.21, *95% CI*: 0.73–2.01, *p* = 0.46), those with lower paternal education levels (e.g., junior high school or below: *OR* = 0.51, *95% CI*: 0.25–1.04, *p* = 0.07; high school/vocational school: *OR* = 0.47, *95% CI*: 0.23–0.96, *p* = 0.04), those who had not heard of HPV (*OR* = 0.45, *95% CI*: 0.30–0.67, *p* < 0.001), those who had not actively searched for or consulted about the HPV vaccine (*OR* = 0.71, *95% CI*: 0.60–0.84, *p* < 0.001), those with higher knowledge levels of HPV (*OR* = 1.06, *95% CI*: 1.03–1.1, *p* < 0.001), those with stronger self-efficacy (*OR* = 0.73, *95% CI*: 0.61–0.88, *p* < 0.001), those with weaker perceived obstacles (*OR* = 1.96 *95% CI*: 1.72–2.24, *p* < 0.001), those with weaker social motivation (*OR* = 0.37, *95% CI*: 0.32–0.44, *p* < 0.001), and those with stronger self-decision-making abilities (*OR* = 1.77, *95% CI*: 1.48–2.11, *p* < 0.001) were significantly more likely to express willingness to vaccinate.

### 3.5. Reasons for Female College Students’ Unwillingness to Receive Vaccinations

**Figure 3:**
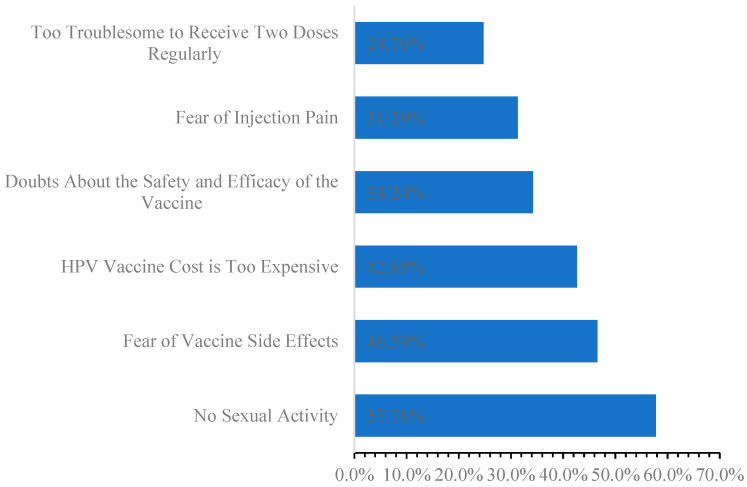
Reasons for female college students’ unwillingness to receive HPV vaccinations.

The most frequently cited reasons included having no sexual activity (57.76%), fear of vaccine side effects (46.59%), and doubts about the vaccine’s safety and efficacy (34.24%). Economic factors, such as the cost of the vaccine (42.65%) and the inconvenience of receiving two doses (24.76%), also played a significant role(Figure 3).

## 4. Discussion

### 4.1. Key Findings and Implications

The low vaccination rate of 18.11% highlights a significant gap in HPV vaccine uptake, despite the availability of effective vaccines. This is consistent with previous studies in China, which have also reported suboptimal HPV vaccination rates among young women [26]. Globally, coverage among female university students varies significantly: Cyprus 30.7% [27], Brazil 31.3% (≥2 doses) [28], and Qatar 6.3% [29]. In contrast, the U.S. achieves higher rates, with 56% initiation and 79% completion [7]. Jordan reports an extremely low rate of 3.6% among female university students in health schools [30]. These disparities highlight the need for targeted strategies to improve global HPV vaccine coverage. The relatively high proportion of students willing to vaccinate (40.19%) suggests that there is potential for increasing vaccination rates through targeted interventions.

Our study found that only 7.11% of female college students reported engaging in sexual activity, a figure significantly lower than the 26.61% reported in a multi-province survey of Chinese adolescents [31] and substantially lower than the approximately 60.8% reported among U.S. college students [12]. This disparity is likely attributable to cultural conservatism, particularly in regions influenced by Confucian values (e.g., Shandong), where premarital sexual activity is stigmatized. These cultural norms may indirectly influence decisions regarding HPV vaccination, as 57.76% of unvaccinated students cited “no sexual activity” as their primary reason, reflecting the misconception that HPV risk is solely associated with sexual behavior. However, HPV transmission through non-sexual means (e.g., skin-to-mucosal contact) is well-documented [32], yet this information is seldom emphasized in health education programs targeting this demographic. In addition to cultural influences, structural and cognitive barriers significantly impede vaccination uptake. Financial limitations (42.65%) and safety concerns (46.59%) reflect challenges commonly encountered in low-resource settings worldwide [33,34]. Importantly, despite World Health Organization (WHO) recommendations that prioritize vaccination for individuals aged 9–14, our study cohort (ages 17–24) constitutes a high-risk group with peak rates of HPV infection, highlighting the critical need for catch-up vaccination programs. Implementing subsidized pricing or government-funded initiatives, as successfully demonstrated in Australia [35], could alleviate economic barriers.

Alarmingly, knowledge gaps exacerbate vaccination hesitancy and cervical cancer screening. Only 17.05% of the participants recognized that early-stage cervical cancer is asymptomatic, while 73.50% erroneously believed that vaccination negates the necessity for cervical screening. These misconceptions indicate a widespread “false sense of security” following vaccination. Furthermore, there is limited awareness of HPV prevention strategies, with only 61.63% acknowledging that condom use reduces transmission, and only 51.62% understanding that vaccination is most effective before sexual debut. These findings underscore systemic deficiencies in sexual health education, as such misinformation is strongly associated with delayed screening and vaccination uptake [36]. This study further revealed that knowledge levels regarding HPV and cervical cancer were significantly higher among the vaccinated group compared to those who were either willing or unwilling to receive the vaccine. This observation aligns with previous research indicating a positive correlation between increased knowledge and higher vaccine uptake [37,38]. However, the overall knowledge scores, particularly concerning cervical cancer, were notably low, highlighting the urgent need for enhanced educational initiatives. Public health campaigns should focus on increasing awareness and understanding of HPV and cervical cancer, especially among populations with lower knowledge levels.

Elevated levels of self-efficacy can enhance an individual’s confidence in their ability to receive vaccination, notwithstanding potential challenges. Our analysis employing the Health Belief Model (HBM) identified significant variations in health beliefs among female college students based on their HPV vaccination status, particularly in terms of perceived susceptibility, perceived benefits, perceived barriers, social motivation, self-decision-making, and self-efficacy (all *p*< 0.001). These dimensions were identified as significant predictors of vaccination status, consistent with prior research [39,40], and they clarify the psychological and social factors influencing HPV vaccination behavior, providing a scientific basis for effective intervention strategies [41]. Notably, perceived severity did not significantly vary (*p* = 0.699), indicating that although students acknowledge the seriousness of HPV-related diseases, they do not act, illustrating the “intention–behavior gap” [42]. This underscores the importance of addressing practical barriers rather than merely enhancing risk communication.

Further correlation analysis revealed that the strong positive relationship between self-efficacy and self-decision-making (*r* = 0.81) suggests that empowering individuals with the skills and confidence to make informed vaccination decisions could increase vaccine uptake. Conversely, the negative correlation between social motivation and self-efficacy (*r* = −0.61) indicates that external pressures may diminish personal agency, especially in collectivist cultures [43]. Interestingly, this study found a negligible negative correlation between perceived benefits and perceived susceptibility (*r* = −0.02), suggesting that students who value vaccination might paradoxically underestimate their own risk. This bias is likely exacerbated by misinformation. These findings underscore the necessity for interventions that address both practical barriers and cognitive biases to enhance HPV vaccine uptake, including perceived benefits and self-efficacy.

This study revealed the complexity of HPV vaccination decisions and willingness to vaccinate among female college students through multiple logistic regression analysis. In the comparison between VG, WTG, and UTG, we found that personal beliefs, such as self-decision-making (VG vs. UTG *OR* = 1.80, *p* < 0.001, WTG vs. UTG *OR* = 1.77, *p* < 0.001) and perceived benefits (VG vs. UTG *OR* = 1.54, *p* < 0.001), were significantly correlated with vaccination intention. However, these positive cognitive factors are often offset by systemic barriers, such as perceived barriers (VG vs. UTG *OR* = 3.34, *p* < 0.001, WTG vs. UTG *OR* = 1.96, *p* < 0.001) and social motivation (VG vs. UTG *OR* = 0.21, *p* < 0.001, WTG vs. UTG *OR* = 0.37, *p* < 0.001). In particular, paternal educational level (VG vs. UTG *OR* = 0.36–0.41, *p* < 0.05) and rural residence (VG vs. UTG *OR* = 0.64, *p* < 0.001) significantly affect vaccination rates, indicating that family dynamics and socioeconomic stratification play important roles in health decision-making [44]. The results indicate that conventional theories of health decision-making, which predominantly emphasize individual knowledge, inadequately account for the discrepancy between the desire for vaccination and actual vaccine uptake among students. Instead, it is crucial to consider the influence of social and economic determinants (e.g., familial educational background) on these decisions [45,46].

Several notable socio-cultural factors were identified, revealing a significant association with reduced vaccination rates (*OR* = 0.64, *p* < 0.001). However, no significant difference was observed in the vaccination rates between VTG and UTG (*OR* = 1.07, *p* = 0.48). Furthermore, a negative correlation was observed between sexual activity history and vaccination (VG: *OR* = 0.57, *p* = 0.01), potentially reflecting societal moral perceptions regarding sexual purity, which can significantly influence an individual’s health behaviors and decision-making. More unexpectedly, students with higher levels of HPV knowledge exhibited greater vaccine hesitancy within WTG (*OR* = 0.45, *p* < 0.001), suggesting that the relationship between knowledge and behavior is not linear but is influenced by the quality of information and methods of dissemination. Contrary to the prevalent assumption that increased knowledge correlates with improved health outcomes, individuals possessing knowledge about HPV (WTG vs. UTG: *OR* = 0.45, *p* < 0.001) or those who actively sought information about the vaccine (VG vs. UTG: *OR* = 0.45, *p* < 0.001) paradoxically demonstrated decreased intention to vaccinate or lower actual vaccination rates. This unexpected pattern may be attributed to the phenomenon of “information overload” and the amplification of perceived risks within the digital health landscape [47]. Specifically, individuals who actively seek information may encounter conflicting narratives or sensationalized accounts of vaccine risks online, resulting in decision paralysis [48]. Similarly, the diminished likelihood of vaccination among those with a family history of cervical cancer (VG vs. UTG: *OR* = 0.37, *p* = 0.01) may be indicative of fatalistic beliefs or genetic determinism, where individuals perceive vaccination as unnecessary due to an assumed hereditary immunity. This cognitive bias has been documented in cancer prevention research [49]. To address these challenges, we propose a series of practical interventions, including public health campaigns that reconceptualize the HPV vaccine as a preventive measure against cancer, rather than merely a choice for sexually active individuals. Establishing an accurate knowledge framework regarding HPV vaccine is essential for enhancing public awareness and acceptance.

### 4.2. Study Limitations

This study has several limitations. This study acknowledges potential biases arising from the use of the Wenjuanxing survey platform, including selection bias due to reliance on digital distribution through WeChat groups. The sample was obtained through convenience sampling from various universities in Jinan, which may restrict the generalizability of the results. Additionally, the cross-sectional design of this study does not allow for the establishment of causal relationships. Furthermore, the dependence on self-reported data can introduce various biases, particularly impacting sensitive subjects, such as premarital sexual behavior. Firstly, recall bias may result in the underreporting of past behaviors. Secondly, within the context of sexual behavior research, social desirability bias may arise, prompting participants to alter their responses to align with societal norms. Future research should aim to address these limitations by employing more diverse sampling methods, utilizing longitudinal study designs, and incorporating objective data collection techniques.

### 4.3. Future Research Directions

To address the existing limitations, future research should incorporate multi-level methodological enhancements to improve the rigor and applicability of the findings. Conducting studies across diverse geographical regions, such as first-tier, second-tier, and third-tier cities, as well as among various socioeconomic groups, can mitigate sampling bias and facilitate the analysis of regional variations in HPV vaccination behaviors. Employing qualitative interviews can help reduce self-report biases. Additionally, the application of emerging methods, such as machine learning analysis, may provide deeper insights into the complex sociocultural factors affecting vaccine decision-making processes.

From the perspective of implementation science, subsequent studies should concentrate on developing targeted intervention frameworks. To address the identified knowledge gaps, multimedia educational resources, including videos and interactive web pages, will be created specifically for young Chinese female college students concerning HPV vaccination. These resources will integrate online courses and offline lectures to provide comprehensive coverage of fundamental information about HPV vaccines, the significance of vaccination, and safety considerations. Furthermore, an in-depth investigation into the perceived barriers and psychological factors affecting HPV vaccination uptake among this demographic will be undertaken. In addition, a multifaceted intervention strategy will be employed, encompassing educational initiatives, psychological support, and the activation of familial and social networks.

## 5. Conclusions

This study highlights the critical role of knowledge, health beliefs, and sociodemographic factors in influencing HPV vaccination behaviors among young female college students in China. Interventions aimed at increasing vaccination rates should prioritize improving knowledge, addressing perceived barriers, and leveraging the influence of familial and social networks. Additionally, it is crucial to tailor these interventions to the specific needs and contexts of different populations. Policymakers should consider implementing strategies to reduce vaccine costs and enhance access to vaccination services, particularly for underserved populations. By addressing these multifaceted factors, we can formulate comprehensive and efficacious interventions that not only augment vaccination rates but also foster long-term public health benefits. Future research should persist in exploring innovative methodologies and collaborations to further enhance vaccination initiatives and address the identified deficiencies in current practices.

## Figures and Tables

**Table 1:** Measurement items for variables across dimensions of the Health Belief Model (HBM).

Dimensions	Serial Numbering	Items
Perceived Severity	M1	I feel anxious when thinking about HPV infection.
	M2	HPV infection is perceived as a serious condition that may affect my academic life.
	M3	A cervical cancer diagnosis would be devastating to me.
	M4	Cervical cancer would have a significant impact on my life.
Perceived Susceptibility	M5	I believe I am at risk of HPV infection.
	M6	I believe I am at risk of developing cervical cancer.
Perceived Benefits	M7	HPV vaccination should be recommended even after sexual initiation.
	M8	HPV vaccination helps protect against HPV infection.
	M9	HPV vaccination can reduce the risk of cervical cancer.
Perceived Barriers	M10	I am skeptical about the safety and efficacy of the HPV vaccine.
	M11	I find it challenging to decide whether to receive the HPV vaccine.
	M12	I am concerned about the pain associated with the HPV vaccine injection.
	M13	The cost of HPV vaccination is perceived as high.
	M14	I am concerned about potential side effects of the HPV vaccine.
Perceived Motivation	M15	Other individuals my age are considering HPV vaccination.
	M16	My family believes I should receive the HPV vaccine.
	M17	My friends believe I should receive the HPV vaccine.
Self-decision-making	B1	Even if the vaccination is expensive, I am confident that I can complete the HPV vaccination.
	B2	Even if the injection is painful, I am confident that I can complete the HPV vaccination.
	B3	Even though I am slightly concerned about potential side effects, I am confident that I can complete the HPV vaccination.
	B4	Even if it means having to receive three doses regularly, I am confident that I can complete the HPV vaccination.
Self-efficacy	B5	I can comfortably discuss the topic of HPV vaccination with my parents/guardians/nurses/doctors.
	B6	I know the vaccination sites or would search online or call to confirm the location
	B7	I am aware that the HPV vaccine is a self-funded vaccine, and the cost is affordable.
	B8	If I need to get vaccinated, I would set reminders on my calendar to ensure I complete all three doses on schedule.

**Table 2:** Variable assignment.

Variables		
Ethnicity	X_1_	Han = 1; Others = 0
Domicile	X_2_	Urban = 1; Village = 0
Religion	X_3_	Yes = 1; No = 0
Age	X_4_	≤20 = 0; >21 = 1
Monthly disposable fund (RMB)	X_5_	<1500 = 0; 1501~ = 1; 2001~ = 2; > 2501 = 3
Whether one of your parents is a healthcare worker	X_6_	Yes = 1; No = 0
Paternal educational level	X_7_	Junior high school and below = 0; High school/vocational and technical schools = 1; University/ junior college = 2; Graduate students and above = 3
Whether there is a family or friend history of cervical cancer	X_8_	Yes = 1; I don’t know/No = 0
Acceptance of premarital sex	X_9_	Yes = 1; No = 0
Whether you have ever engaged in sexual activity	X_10_	Yes = 1; No = 0
Whether you have received sex education	X_11_	Yes = 1; No = 0
Whether you have ever heard of HPV	X_12_	Yes = 1; No = 0
Whether you have ever actively searched for or consulted about the HPV vaccine	X_13_	Yes = 1; No = 0
Whether the knowledge of HPV is adequate	X_14_	≥7 = 1; <7 = 0
Perceived severity	X_15_	Continuous variables
Perceived susceptibility	X_16_	Continuous variables
Perceived benefits	X_17_	Continuous variables
Perceived barriers	X_18_	Continuous variables
Social motivation	X_19_	Continuous variables
Self-decision-making	X_20_	Continuous variables
Self-efficacy	X_21_	Continuous variables

**Table 3:** Vaccination status of female university students with different demographic characteristics (n = 4076).

Variables	Total (N = 4076, %)	VG (N = 738, %)	WTG (N = 1638, %)	UTG (N = 1700, %)	χ2	*p* Values
Total (n, %)						
Ethnicity					0.745	0.689
Han	3945 (96.78)	717 (97.15)	1581 (96.52)	1647 (96.88)		
Others	131 (3.21)	21 (2.85)	57 (3.48)	55 (3.21)		
Domicile					188.449	<0.001
Urban	1431 (35.11)	417 (56.50)	535 (32.66)	479 (28.18)		
Village	2645 (64.89)	321 (43.50)	1103 (67.34)	1221 (71.65)		
Religion					2.336	0.311
Yes	46 (1.15)	8 (1.08)	14 (0.85)	24 (1.41)		
No	4030 (98.87)	730 (98.92)	1624 (99.15)	1686 (98.59)		
Age					95.534	<0.001
≤20	3591 (88.10)	574 (77.78)	1499 (91.51)	1518 (89.29)		
>21	485 (11.90)	164 (22.22)	139 (8.49)	182 (10.71)		
Monthly disposable fund (RMB)					151.303	<0.001
≤1500	1971 (48.36)	232 (31.44)	787 (48.05)	952 (56.00)		
1501–2000	1482 (36.36)	317 (42.95)	627 (38.28)	538 (31.65)		
2001–2500	402 (9.86)	116 (15.72)	143 (8.73)	143 (8.41)		
>2500	221 (5.42)	73 (9.89)	81 (4.95)	67 (3.94)		
Whether one of your parents is a healthcare worker					39.733	<0.001
Yes	124 (3.04)	48 (6.50)	39 (2.38)	37 (2.18)		
No	3952 (96.96)	690 (93.50)	1599 (97.62)	1663 (97.82)		
Paternal educational level					200.057	<0.001
Junior high school and below	1467 (35.99)	156 (21.14)	611 (37.30)	700 (41.18)		
High school (including vocational and technical schools)	1483 (36.38)	234 (31.71)	604 (36.87)	645 (37.94)		
University (including junior college and undergraduate)	1053 (25.83)	323 (43.77)	390 (23.81)	340 (20.00)		
Graduate students and above	73 (1.79)	25 (3.39)	33 (2.01)	15 (0.88)		
Acceptance of premarital sex					73.003	<0.001
Yes	1681 (41.24)	392 (53.12)	697 (42.55)	592 (34.82)		
No	2395 (58.76)	346 (46.88)	941 (57.45)	1108 (65.18)		
Whether you have ever engaged in sexual activity					55.079	<0.001
Yes	290 (7.11)	99 (13.41)	101 (6.17)	90 (5.29)		
No	3786 (92.89)	639 (86.59)	1537 (93.83)	1610 (94.71)		
Whether there is a family or friend history of cervical cancer					66.918	<0.001
Yes	71 (1.74)	23 (3.12)	27 (1.65)	21 (1.24)		
No	3292 (80.77)	621 (84.15)	1380 (84.25)	1291 (75.94)		
I don’t know	713 (17.49)	94 (12.74)	231 (14.10)	388 (22.82)		
Whether you have received sex education					30.755	<0.001
Yes	3567 (87.51)	676 (91.60)	1458 (89.01)	1433 (84.29)		
No	509 (12.49)	62 (8.40)	180 (10.99)	267 (15.71)		
Whether you have ever heard of HPV					69.060	<0.001
Yes	3864 (94.80)	712 (96.48)	1598 (97.56)	1544 (91.41)		
No	212 (5.20)	26 (3.52)	40 (2.44)	146 (8.59)		
Whether you have ever actively searched for or consulted about the HPV vaccine					325.771	<0.001
Yes	2563 (62.88)	619 (83.88)	1128 (68.86)	816 (48.00)		
No	1513 (37.12)	119 (16.12)	510 (31.14)	884 (52.00)		

**Table 4:** Differences in Health Belief Model (HBM) constructs for vaccination status of female university students.

Variables	Total (N = 4076)	VG (N = 738)	WTG (N = 1638)	UTG (N = 1700)	*F*	*p* Values
Knowledge	5.659 ± 2.975	6.664 ± 2.670	6.135 ± 2.704	4.762 ± 3.104	150.413	<0.001
Knowledge of HPV	4.915 ± 2.459	5.745 ± 2.167	5.316 ± 2.201	4.169 ± 2.606	152.566	<0.001
Knowledge of cervical cancer	0.743 ± 0.846	0.918 ± 0.886	0.819 ± 0.856	0.594 ± 0.793	50.025	<0.001
Motivation						
Perceived severity	2.121 ± 0.851	2.140 ± 0.906	2.109 ± 0.851	2.123 ± 0.825	0.358	0.699
Perceived susceptibility	3.826 ± 1.069	3.919 ± 1.056	3.773 ± 1.074	3.836 ± 1.069	4.856	0.008
Perceived benefits	1.910 ± 0.746	1.736 ± 0.754	1.785 ± 0.676	2.105 ± 0.761	106.317	0.000
Perceived obstacles	2.952 ± 0.773	3.33 ± 0.805	2.986 ± 0.771	2.756 ± 0.691	155.840	<0.001
Social motivation	2.392 ± 0.735	1.925 ± 0.686	2.257 ± 0.672	2.724 ± 0.657	2.392 ± 0.735	<0.001
Behavioral skills						
Self-decision-making	3.801 ± 0.789	4.275 ± 0.733	3.95 ± 0.696	3.452 ± 0.742	391.962	<0.001
Self-efficacy	3.768 ± 0.754	4.24 ± 0.727	3.857 ± 0.691	3.477 ± 0.696	328.42	<0.001

## Data Availability

The raw data supporting the conclusions of this article will be made available by the authors upon request.

## Supplementary Materials

The following supporting information can be downloaded at: https://www.mdpi.com/article/10.3390/vaccines13060598/s1, Table S1. Factors Associated with HPV Vaccination Status.

## References

[1] Wu J., Jin Q., Zhang Y., Ji Y., Li J., Liu X., Duan H., Feng Z., Liu Y., Zhang Y. (2025). Global burden of cervical cancer: Current estimates, temporal trend and future projections based on the GLOBOCAN 2022. J. Natl. Cancer Cent..

[2] Plotzker R.E., Vaidya A., Pokharel U., Stier E.A. (2023). Sexually Transmitted Human Papillomavirus: Update in Epidemiology, Prevention, and Management. Infect. Dis. Clin. N. Am..

[3] Zhang L., Zhao Y., Tu Q., Xue X., Zhu X., Zhao K.-N. (2021). The Roles of Programmed Cell Death Ligand-1/ Programmed Cell Death-1 (PD-L1/PD-1) in HPV-induced Cervical Cancer and Potential for their Use in Blockade Therapy. Curr. Med. Chem..

[4] Włoszek E., Krupa K., Skrok E., Budzik M.P., Deptała A., Badowska-Kozakiewicz A. (2025). HPV and Cervical Cancer—Biology, Prevention, and Treatment Updates. Curr. Oncol..

[5] Kjaer S.K., Nygård M., Sundström K., Dillner J., Tryggvadottir L., Munk C., Berger S., Enerly E., Hortlund M., Ágústsson Á.I. (2020). Final analysis of a 14-year long-term follow-up study of the effectiveness and immunogenicity of the quadrivalent human papillomavirus vaccine in women from four nordic countries. EClinicalMedicine.

[6] Simms K.T., Steinberg J., Caruana M., Smith M.A., Lew J.B., Soerjomataram I., Castle P.E., Bray F., Canfell K. (2019). Impact of scaled up human papillomavirus vaccination and cervical screening and the potential for global elimination of cervical cancer in 181 countries, 2020–2099: A modelling study. Lancet Oncol..

[7] Alsulami F.T., Sanchez J., Rabionet S.E., Popovici I., Baraka M.A. (2023). Predictor of HPV Vaccination Uptake among Foreign-Born College Students in the U.S.: An Exploration of the Role of Acculturation and the Health Belief Model. Vaccines.

[8] Liu Y., Di N., Tao X. (2020). Knowledge, practice and attitude towards HPV vaccination among college students in Beijing, China. Hum. Vaccin. Immunother..

[9] Song D., Liu P., Wu D., Zhao F., Wang Y., Zhang Y. (2023). Knowledge and Attitudes towards Human Papillomavirus Vaccination (HPV) among Healthcare Providers Involved in the Governmental Free HPV Vaccination Program in Shenzhen, Southern China. Vaccines.

[10] Zhao X.L., Hu S.Y., Hu J.W., Wang H.H., Wen T.M., Feng Y.S., Qiao Y.L., Zhao F.H., Zhang Y. (2023). Tackling barriers to scale up human papillomavirus vaccination in China: Progress and the way forward. Infect. Dis. Poverty.

[11] Wang S., Wu H., Zhang H., Niu X., Pan L., Wang X., Qin W., Hu F., Li L., Yang H. (2024). Gender differences in sexual health knowledge, attitude and behavior among Chinese college students and the influencing factors of sexual behavior. Chin. J. Dis. Control Prev..

[12] Azim K.A., Happel-Parkins A., Moses A., Haardoerfer R. (2021). Exploring Relationships Between Genito-Pelvic Pain/Penetration Disorder, Sex Guilt, and Religiosity Among College Women in the U.S. J. Sex. Med..

[13] Hansen B.T., Kjær S.K., Arnheim-Dahlström L., Liaw K.L., Juul K.E., Thomsen L.T., Frederiksen K., Elfström K.M., Munk C., Nygård M. (2020). Age at first intercourse, number of partners and sexually transmitted infection prevalence among Danish, Norwegian and Swedish women: Estimates and trends from nationally representative cross-sectional surveys of more than 100,000 women. Acta Obs. Gynecol. Scand..

[14] Hu S., Xu X., Zhang Y., Liu Y., Yang C., Wang Y., Wang Y., Yu Y., Hong Y., Zhang X. (2021). A nationwide post-marketing survey of knowledge, attitude and practice toward human papillomavirus vaccine in general population: Implications for vaccine roll-out in mainland China. Vaccine.

[15] Zhang X., Liu C.R., Wang Z.Z., Ren Z.F., Feng X.X., Ma W., Gao X.H., Zhang R., Brown M.D., Qiao Y.L. (2020). Effect of a school-based educational intervention on HPV and HPV vaccine knowledge and willingness to be vaccinated among Chinese adolescents: A multi-center intervention follow-up study. Vaccine.

[16] Glanz K., Rimer B.K., Lewis F.M. (1997). Health Behavior and Health Education: Theory, Research, and Practice.

[17] Rosenstock I.M. (1974). Historical Origins of the Health Belief Model. Health Educ. Monogr..

[18] Finfgeld D.L., Wongvatunyu S., Conn V.S., Grando V.T., Russell C.L. (2010). Health Belief Model and Reversal Theory: A comparative analysis. J. Adv. Nurs..

[19] Krebs P., Prochaska J.O., Rossi J.S. (2010). A meta-analysis of computer-tailored interventions for health behavior change. Prev. Med..

[20] Jinan Municipal Government Jinan, a City of Springs Overview[EB/OL]. (25 February 2025). http://english.jinan.gov.cn/col/col108341/index.html.

[21] China Daily Jinan Honored as ‘2023 Annual Dynamic City’ [EB/OL]. (28 December 2023). http://shandong.chinadaily.com.cn/2023-12/28/c_951937.htm.

[22] Dang J.H., Stewart S.L., Blumberg D.A., Rodriguez H.P., Chen M.S. (2021). Patient and clinician factors associated with uptake of the human papillomavirus (HPV) vaccine among adolescent patients of a primary care network. Vaccine.

[23] Dai Z., Si M., Su X., Wang W., Zhang X., Gu X., Ma L., Li J., Zhang S., Ren Z. (2022). Willingness to human papillomavirus (HPV) vaccination and influencing factors among male and female university students in China. J. Med. Virol..

[24] Hui C. (2021). Evaluation of a Web-Based Health Education Based on IMB Model in Improving Female College Students’ Knowledge on HPV Vaccines and Their Willingness to be Vaccinated.

[25] Si M. (2021). An IMB Model-Based Study on Influencing Factors and Intervention on HPV Vaccination Among College Girls.

[26] Bai Y., Ip P., Chan K., Ngan H., Yip P. (2022). HPV Vaccination Intentions of Female Students in Chinese Universities: A Systematic Literature Review and Meta-Analysis. Int. J. Environ. Res. Public Health.

[27] Charalambous I., Ioannou G., Nikolaou S., Theologou R., Yiallourou A., Papatheodorou S., Pantavou K.G., Nikolopoulos G.K. (2020). State of knowledge of human papillomavirus (HPV), HPV vaccine and testing: A cross-sectional study among female university students in Cyprus. Women Health.

[28] Oliveira P.S.D., Gonçalves C.V., Watte G., Costa J.S.D.D. (2021). Vaccination coverage against human papillomavirus (HPV) and associated factors in female academics from a university in southwestern Goiás, Brazil. Rev. Saude Publica.

[29] Cheema S., Abraham A., Maisonneuve P., Jithesh A., Chaabna K., Al Janahi R., Sarker S., Hussain A., Rao S., Lowenfels A.B. (2024). HPV infection and vaccination: A cross-sectional study of knowledge, perception, and attitude to vaccine uptake among university students in Qatar. BMC Public Health.

[30] Sallam M., Al-Mahzoum K., Eid H., Assaf A.M., Abdaljaleel M., Al-Abbadi M., Mahafzah A. (2021). Attitude towards HPV Vaccination and the Intention to Get Vaccinated among Female University Students in Health Schools in Jordan. Vaccines.

[31] Zhao R., Zhang L. (2019). Sexual and reproductive health related knowledge, attitude and behavior among senior high school and college students in 11 provinces and municipalities of China. Chin. J. Public Health.

[32] Ardekani A., Taherifard E., Mollalo A., Hemadi E., Roshanshad A., Fereidooni R., Rouholamin S., Rezaeinejad M., Farid-Mojtahedi M., Razavi M. (2022). Human Papillomavirus Infection during Pregnancy and Childhood: A Comprehensive Review. Microorganisms.

[33] Drolet M., Laprise J.-F., Martin D., Jit M., Bénard É., Gingras G., Boily M.-C., Alary M., Baussano I., Hutubessy R. (2021). Optimal human papillomavirus vaccination strategies to prevent cervical cancer in low-income and middle-income countries in the context of limited resources: A mathematical modelling analysis. Lancet Infect. Dis..

[34] Gostin L., Hodge J.G., Bloom B.R., El-Mohandes A., Fielding J., Hotez P., Kurth A., Larson H.J., A Orenstein W., Rabin K. (2020). The public health crisis of underimmunisation: A global plan of action. Lancet Infect. Dis..

[35] NSW Health Human Papillomavirus (HPV) Vaccine-Parent Information [EB/OL]. (13 March 2024) [3/5]. https://www.health.nsw.gov.au/immunisation/Pages/parent-info-hpv.aspx.

[36] Kim S.J., Schiffelbein J.E., Imset I., Olson A.L. (2022). Countering Antivax Misinformation via Social Media: Message-Testing Randomized Experiment for Human Papillomavirus Vaccination Uptake. J. Med. Internet Res..

[37] Bracko M., Simon U.K. (2022). Virus-related Knowledge in Covid-19 Times—Results from two Cross-sectional Studies in Austria and Implications for School. Int. J. Biol. Sci..

[38] Gong X., Xu J., He Y., Zou G., Liu J. (2024). Socioeconomic inequalities in human papillomavirus knowledge and vaccine uptake: Evidence from a cross-sectional study in China. Front. Public. Health.

[39] Getachew T., Negash A., Degefa M., Lami M., Balis B., Debela A., Gemechu K., Shiferaw K., Nigussie K., Bekele H. (2023). COVID-19 vaccine acceptance and associated factors among adult clients at public hospitals in eastern Ethiopia using the health belief model: Multicentre cross-sectional study. BMJ Open.

[40] Han Y., Yin J., Zeng Y., Chu C.-I., Chiang Y.-C., Fang Y. (2019). Determinants of Parental Intentions to Vaccinate Kindergarten Children Against Seasonal Influenza in Xiamen, China. J. Prim. Prev..

[41] Zomordi G., Moradi M., Hasanzadeh M., Ghavami V. (2022). The effect of education based on the theory of planned behavior on the intention of vaccination against human papillomavirus in female students: A controlled educational trial. J. Educ. Health Promot..

[42] Shmueli L. (2021). Predicting intention to receive COVID-19 vaccine among the general population using the health belief model and the theory of planned behavior model. BMC Public Health.

[43] Ma A., Savani K., Liu F., Tai K., Kay A.C. (2023). The mutual constitution of culture and psyche: The bidirectional relationship between individuals’ perceived control and cultural tightness-looseness. J. Pers. Soc. Psychol..

[44] Zhou W., Hou J., Sun M., Wang C. (2022). The Impact of Family Socioeconomic Status on Elderly Health in China: Based on the Frailty Index. Int. J. Environ. Res. Public Health.

[45] Glassman L.W., Szymczak J.E. (2022). The influence of social class and institutional relationships on the experiences of vaccine-hesitant mothers: A qualitative study. BMC Public Health.

[46] Umman V., Girgin T., Baki B.E., Bozbiyik O., Akbulut S., Yoldas T. (2024). Impact of pandemic and socioeconomic influences on decision-making for emergency ostomy procedures: Key factors affecting hospital visit decisions. Medicine.

[47] Hatfield E., Berube D.M. (2024). Information Overload and the COVID-19 Vaccine. Pandemic Resilience: Vaccination Resistance and Hesitance, Lessons from COVID-19.

[48] Singh L., Bao L., Bode L., Budak C., Pasek J., Raghunathan T., Traugott M., Wang Y., Wycoff N. (2024). Understanding the rationales and information environments for early, late, and nonadopters of the COVID-19 vaccine. Npj Vaccines.

[49] Chen X., Wang L., Huang Y., Zhang L. (2024). Risk perception and trust in the relationship between knowledge and HPV vaccine hesitancy among female university students in China: A cross-sectional study. BMC Public Health.

