# Supplementary material for: Coverage of HPV Vaccination and Influencing Factors Among Female College Students in Northern China
Source: Vaccines (Basel). 2025 May 31;13(6):598. doi: 10.3390/vaccines13060598 (PMC12197517; doi:10.3390/vaccines13060598)
Supplement: Supplementary file 1 [file vaccines-13-00598-s001.zip › vaccines-3579243-supplementary.pdf]

Table S1. Factors Associated with HPV Vaccination Status

Variables	B	Wald	OR (95% CI)	P	B	Wald	OR (95% CI)	P
Ethnicity(Others Vs Han)	-0.05	0.02	0.95(0.52,1.75)	0.88	0.15	0.48	1.16(0.76,1.78)	0.49
Domicile(Rural Vs Urban)	-0.45	11.88	0.64(0.49,0.82)	<0.001	0.07	0.51	1.07(0.88,1.3)	0.48
Religion(No Vs Yes)	-0.26	0.28	0.77(0.3,2)	0.60	0.33	0.83	1.4(0.68,2.86)	0.36
Age(≤20 years old Vs >21 years old)	-0.96	41.09	0.38(0.28,0.51)	<0.001	0.36	7.49	1.43(1.11,1.85)	**0.01**
Living expenses (Yuan/Month)								
1≤1500 Vs >2501	-0.32	1.84	0.72(0.45,1.16)	0.17	-0.05	0.05	0.96(0.64,1.42)	0.82
1501-2000 Vs >2501	0.28	1.45	1.32(0.84,2.09)	0.23	0.23	1.28	1.25(0.85,1.86)	0.26
2001-2500 Vs >2501	0.10	0.15	1.11(0.66,1.86)	0.70	0	0	1.00(0.64,1.57)	0.99
Whether one of your parents is a healthcare worker(No Vs Yes)	-0.18	0.41	0.83(0.47,1.46)	0.52	0.19	0.55	1.21(0.73,2.01)	0.46
Paternal educational level								
Junior high school and below Vs Graduate students and above	-1.03	5.97	0.36(0.16,0.82)	0.01	-0.67	3.39	0.51(0.25,1.04)	0.07

High school /vocational and technical schools Vs Graduate students and above	-0.9	4.78	0.41(0.18,0.91)	0.03	-0.75	4.34	0.47(0.23,0.96)	0.04
University/ junior college Vs Graduate students and above	-0.33	0.66	0.72(0.33,1.59)	0.42	-0.61	2.91	0.54(0.27,1.09)	0.09
Whether there is a family or friend history of cervical cancer.(No/I don't know Vs Yes)	-1.00	7.34	0.37(0.18,0.76)	0.01	-0.43	1.78	0.65(0.34,1.22)	0.18
Acceptance of premarital sex (No Vs Yes)	-0.13	1.24	0.88(0.70,1.10)	0.27	-0.04	0.2	0.96(0.81,1.14)	0.66
Whether you have ever engaged in sexual activity.(No Vs Yes)	-0.55	7.67	0.57(0.39,0.85)	0.01	0.04	0.06	1.04(0.74,1.46)	0.81
Whether you have received sex education .(No Vs Yes)	0.25	1.66	1.28(0.88,1.86)	0.20	0.19	2.49	1.21(0.95,1.54)	0.11
Whether you have ever heard of HPV.(No Vs Yes)	0.09	0.11	1.1(0.64,1.9)	0.74	-0.8	15.75	0.45(0.3,0.67)	<0.001
Whether you have ever actively searched for or consulted about the HPV vaccine.(No Vs Yes)	-0.80	33.77	0.45(0.34,0.59)	<0.001	-0.34	15.94	0.71(0.6,0.84)	<0.001
Knowledge level of HPV.	0.03	2.14	1.03(0.99,1.08)	0.14	0.06	18.14	1.06(1.03,1.1)	<0.001
Perceived Severity	0.08	1.28	1.08(0.94,1.25)	0.26	0.09	2.92	1.10(0.99,1.22)	0.09
Perceived susceptibility	-0.05	1.01	0.95(0.85,1.05)	0.31	-0.08	3.79	0.93(0.86,1.00)	0.05
Perceived benefits	0.43	19.9	1.54(1.27,1.86)	<0.001	-0.05	0.58	0.95(0.82,1.09)	0.45
Perceived obstacles	1.21	193.39	3.34(2.82,3.96)	<0.001	0.67	98.33	1.96(1.72,2.24)	<0.001

Social Motivation	-1.57	197.04	0.21(0.17,0.26)	<0.001	-0.99	147.41	0.37(0.32,0.44)	<0.001
Self decision making	0.59	18.88	1.8(1.38,2.35)	<0.001	0.57	39.01	1.77(1.48,2.11)	<0.001
Self efficacy	0.18	1.79	1.2(0.92,1.57)	0.18	-0.31	10.9	0.73(0.61,0.88)	<0.001

Abbreviations: VG = Vaccinated Group; UTG = Unwilling-to-Vaccinate Group; WTG = Willing-to-Vaccinate Group; OR = Odds Ratio; CI = Confidence Interval.
